# Improving Glass Transition Temperature and Toughness of Epoxy Adhesives by a Complex Room-Temperature Curing System by Changing the Stoichiometry

**DOI:** 10.3390/polym15020252

**Published:** 2023-01-04

**Authors:** Oiane Ruíz de Azúa, Núria Agulló, Jordi Arbusà, Salvador Borrós

**Affiliations:** 1 Grup d’Engineyeria de Materials (GEMAT), Institut Quimic de Sarria (IQS), Universitat Ramon Lull, 08017 Barcelona, Spain; 2 Sailing Techcnologies, S.L., 08017 Barcelona, Spain

**Keywords:** epoxy adhesives, glass transition temperature, room-temperature curing, mechanical properties, stoichiometry

## Abstract

The glass transition temperature (Tg) of room-temperature curing epoxy adhesives is limited by the temperature used during curing. It is already known that the excess of epoxy groups can undergo a homopolymerization reaction initiated by tertiary amines at elevated temperatures, resulting in an increase in Tg. However, there is no evidence of this reaction occurring at room temperature. In the present work, the influence of formulation stoichiometry on Tg and mechanical properties was investigated. Dynamomechanical, rheological and mechanical properties of epoxy adhesives were determined by DSC, DMA, rheometer and tensile and shear strength testing. It has been probed that an excess of epoxy resin combined with a complex curing system composed of a primary amine, a polymercaptan and a tertiary amine leads to an increase in Tg up to 70 °C due to the homopolymerization reaction that takes place at room temperature. However, as the excess of epoxy resin is increased, gel time becomes slower. Regarding mechanical properties, it has been proven that an excess of epoxy resin provides a tighter and tougher material but maintains flexibility of the stoichiometric formulation, which is meant to enhance the resistance to impact-type forces, thermal shock and thermal cycling.

## 1. Introduction

Epoxy resin is a type of reactive prepolymer and polymer containing epoxide groups. This material has many industrial applications for a variety of purposes [1,2,3,4,5,6]. It possesses higher mechanical properties and more thermal and chemical resistance than other types of resin [7,8]. These resins react either with themselves in the presence of catalysts, or with many co-reactants such as amines, phenols and thiols, among others [9,10]. These materials are of great importance in the adhesives field. Epoxy resins can be modified and formulated with a wide variety of additives, giving rise to a very versatile family of adhesives [11]. Nowadays, a wide variety of epoxy adhesives that meet different requirements are available in the market.

Since epoxy adhesive systems are mainly used for structural purposes, one of the properties to be considered is the glass transition temperature (Tg). Tg is the temperature at which the transition between the glassy and rubbery state of amorphous solids occurs. Above this value, the polymer behaves like a rubbery material, and below this value, the polymer chains have relatively low mobility, and the material becomes hard and rigid. Thus, Tg it is considered an extremely useful yardstick for the reliability of this type of structural adhesive.

There are several factors contributing to Tg of epoxy adhesives: the chemical structure of epoxy resin, the type of hardener, the degree of cure and the curing conditions (time and temperature (Tcure)) [12,13,14,15,16,17,18,19]. Many studies have been conducted to evaluate the relationship between Tcure and Tg [20,21,22,23]. Some have been focused on the time–temperature–transition (TTT) diagrams of thermoset systems, particularly those cured by the step polymerization mechanism [24,25,26,27,28]. Oleinik [29] on the other hand, reported that in aromatic-amine epoxy systems, the curing temperature and the glass transition temperature can be related by Equation (1). Their studies concluded that the resulting Tg could not be more than 15–30 °C higher than Tcure.

Tg ≈ Tcure + 0.5 ΔTg(1)

This work led to a long-established rule of thumb that Tg cannot exceed Tcure by more than 25 °C. That is the reason why commercial room-temperature curing epoxy adhesives always have a Tg limited to 40–50 °C. On the other hand, high-temperature curing epoxy adhesives can reach values up to 160 °C [30]. The higher the Tg, the better performance for these structural adhesive systems; however, high curing temperature cannot be applied to every substrate. Consequently, the scientific challenge consists of formulating an adhesive system that can achieve high Tg when curing at room temperature.

Regarding this matter, Fernandez-Francos et al. studied a new dual-cure system based on epoxy/thiol formulations catalyzed by tertiary amines [31]. The study concluded that in the presence of excess epoxy groups, thiol-epoxy condensation took place in the first stage of curing, while excess epoxy homopolymerized in the second stage once the thiol groups were depleted, thus increasing the Tg of the formulation. However, all these studies were carried out by high-temperature curing or postcuring processes. Therefore, there is no evidence to conclude whether this polymerization process also takes place at room temperature.

In order to study a possible approach to increase the Tg of room-temperature curing epoxy adhesive formulations without the use of an external heat source, a new formulation has been proposed. This new formulation consists of a on a nonstoichiometric epoxy adhesive using a complex curing based on a commercial adhesive developed by Dr. Sails^®^.

## 2. Materials and Methods

### 2.1. Chemicals

**Figure 1:**
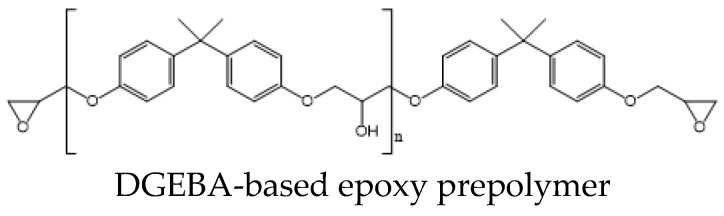
Chemical structures of epoxy prepolymer.

Diglycidyl ether of bisphenol-A (DGEBA) (Araldite^®^ PY 302-2, Huntsman, Pamplona, Spain) with an equivalent molecular weight of 170 g/eq was used as the base epoxy resin. It is a highly crystallization-resistant, low-viscosity liquid epoxy resin with superior chemical resistance. It presents a viscosity between 6500–8000 mPa-s^−1^ and a density of 1.17 g/cm^3^. The basic chemical structure of epoxy resin used in this investigation is shown in Figure 1. Aradur^®^ 90, Hunstman, Pamplona, Spain polymercaptan with tertiary amine was also supplied by Huntsman. It presents a viscosity between 10,000–160,000 mPa.s^−1^, a H^+^ active equivalent of 200 g/Eq and a gel time against Araldite GY 302-2 of 4–5 min. Ethylenediamine was purchased from Aldrich. All chemicals were used as received.

Dr. Sails^®,^ Sailing Technologies, Barcelona, Spain commercial adhesive was supplied by Sailing Technologies S.L. Dr. Sails^®^ is a commercial two-component adhesive capable of curing underwater. The main chemical in component 1 is Araldite PY 302-2^®^ and the main chemical in component 2 is Aradur 90^®^. It was designed to be used in a 1:1 proportion.

### 2.2. Formulation and Cure Schedules

Adhesive formulations were prepared by mixing Dr. Sails^®^ commercial epoxy adhesive epoxy at fixed ratios shown in Table 1.

### 2.3. Kinetic Analysis of the Curing Process Using Differential Scanning Calorimetry (DSC)

Calorimetric studies were carried out using Mettler Toledo DSC equipment, Barcelona, Spain. Samples were analyzed using perforated aluminum capsules under an inert atmosphere (N_2_) with a flow rate of 50 mL/min. Dynamic experiments to determine the curing enthalpies of the prepared mixtures were carried out at a heating rate of 10 °C/min between 30 °C and 300 °C.

### 2.4. Curing Time Analysis

Curing time characterization was made with a AR2000 rheometer (TA Instrument), Cerdanyola, Spain using a 20 mm steel plate. The gap was set at 300 microns. An oscillatory procedure was applied using a constant strain of 5% at 1 Hz. Analyses were carried out isothermally at 25 °C.

Gel time was determined as the crossing between the G’ and G’’ curves; at this point, the system stores the same or a very similar amount of energy to that dissipated.

### 2.5. Viscoelastic Properties of the Cured Material

A TA instrument dynamic mechanical analyzer (DMA) Q800 apparatus (TA Instruments, Cerdanyola, Spain) was used in this study, along with a 3-point bending clamp. Tested specimens were rectangular-shaped, 60 mm × 15 mm × 2 mm. The linear viscoelastic region (LVR) of the material was determined by an amplitude sweep test, from 10 to 60 μm at a constant temperature of 25 °C. Tg was determined using a temperature ramp test from 30 to 100 °C with a heating rate of 5 °C/min, an amplitude of 30 μm and a frequency of 1 Hz. The tan δ peak was used as a viscoelastic parameter to calculate the Tg of each formulation.

### 2.6. Tensile Testing

Tensile tests were conducted following ASTM D638 standard protocol [32] and using ZwickRoell Z030 equipment, Sant Cugat del Vallès, Spain. Complete curing of the adhesive was achieved by keeping the samples at room temperature for one week. Probes were prepared with a 4 mm thickness. Results of maximum tensile strength were reported.

### 2.7. Shear Testing

UNE-EN 22643 test standard protocol [33] was followed for shear testing. Assays were performed using ZwickRoell Z030 equipment, Sant Cugat del Vallès, Spain. Complete curing of the adhesive was achieved by keeping the samples at room temperature for one week. The adhesive thickness of the bonded specimens was 1.5 mm. Results of tensile shear strength were reported.

## 3. Results and Discussion

Before studying the curing mechanism, the curing behavior of epoxy was analyzed by FTIR. The FTIR spectra are presented as Supplementary Information (Figures S1–S4). Briefly, two characteristic absorptions of the oxirane ring of epoxy were observed in the range between 3500 cm^−1^ and 700 cm^−1^. The first one, at 911 cm^−1^, is attributed to the C-O deformation of the oxirane group. The second band is located at approximately 3057 cm^−1^ and is attributed to the C-H tension of the methylene group in the epoxy ring, although it is also related to asymmetrical and symmetrical C-H stretch of aromatic ring. The presence of these two peaks at the spectre states that the epoxy resin has not reacted yet. However, once the epoxy resin starts curing, the oxirane ring concentration decreases, and this was observed in the spectra as the decrease in the two characteristic absorption bands until all oxirane rings are depleted, as can be seen in Figures S2–S4.

### 3.1. Kinetic Analysis of Curing Process by DSC

Kinetic analysis of the reactions that take part in the studied systems were analyzed by DSC. The aim of the present study is to develop a complex curing system able to generate enough heat to activate the homopolymerization of the excess of epoxy resin.

**Figure 2:**
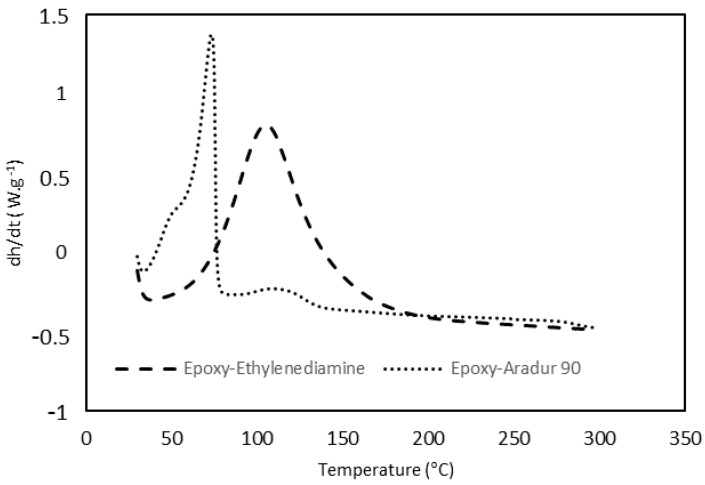
Curing schedule analysis by DSC for epoxy-amine and epoxy-thiol.

Stoichiometric mixtures of Araldite^®^PY302-2 epoxy resin with ethylenediamine on one hand and Araldite^®^PY 302-2 epoxy resin with Aradur^®^90 on the other hand were studied in order to determine characteristic exotherm peaks of epoxy-amine and epoxy-thiol crosslinking reactions. As shown in Figure 2, the first exothermic peak (A) that appears around 80 °C corresponds to the crosslinking reaction between epoxy and thiol crosslinker. A second exothermic transition (B) was observed at around 120 °C, probably due to the presence of tertiary amines in the curing system. However, it seems that the heat released by this reaction is negligible compared to the heat released in the first peak; thus, it can be considered a secondary reaction. The crosslinking reaction described by the epoxy formulated with amine crosslinkers (curve (C) in Figure 2) presents a significant exothermic maximum peak at around 120 °C.

**Figure 3:**
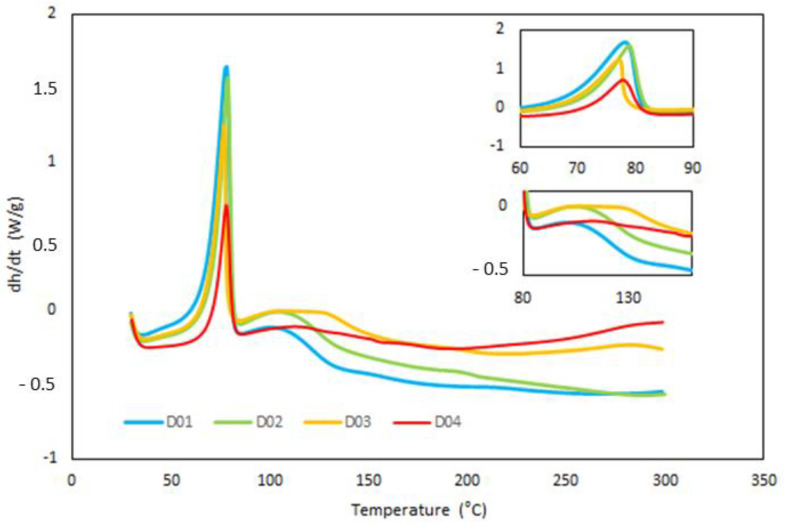
Curing schedule results by DSC analysis.

Once the curing profiles of the reactions involved in the curing of epoxy systems were described and identified, different formulations (D01, D02, D03, D04) were studied in order to determine differences in between stoichiometric and nonstoichiometric formulation reactions (Figure 3).

**Scheme 1:**
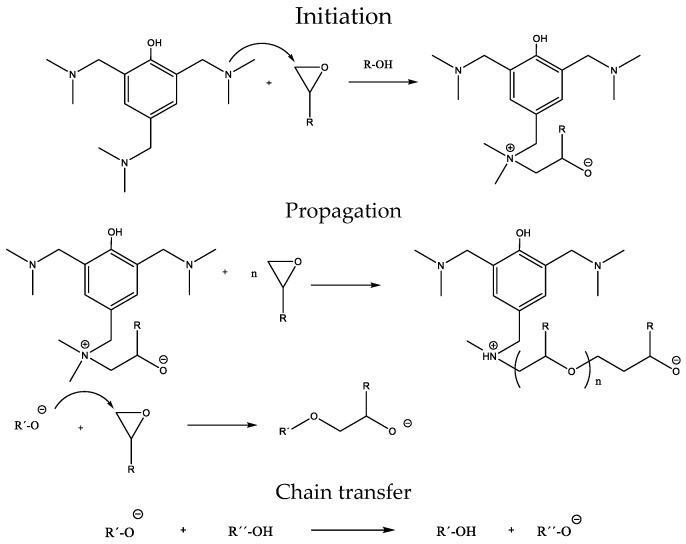

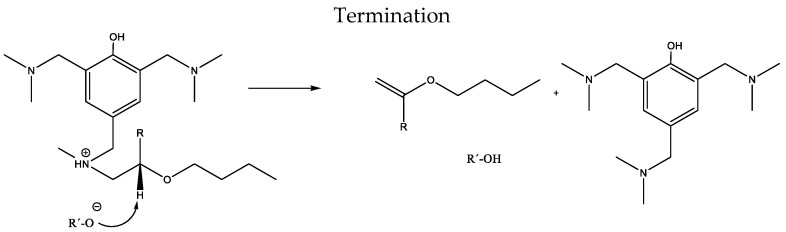
Homopolymerization of epoxy excess by a tertiary amine.

The results obtained showed the different reactions occurring for each tested formulation. When the ratio between the crosslinker’s active groups and epoxy groups is stoichiometric (D01), two separated reaction peaks are detected. The first one corresponds to the reaction between the epoxy and thiol crosslinkers, and the second to the reaction with the amine crosslinkers. As the epoxy ratio in the formulation increases, the appearance of a third peak is detected at around 130 °C, overlapped with the amine curing peak. This phenomenon indicates that a third reaction is taking place during the curing, and such a reaction can only be due to the homopolymerization of the excess of DGEBA (as indicated in Scheme 1). There are no other compounds in the formulation able to give rise to a reaction.

The overall heat released during the whole curing process of D01, D02 and D03 formulations is higher than 170 J·g^−1^, except for the D04 formulation, which does not even reach the 100 J·g^−1^ (Table 2). The higher the ratio of DGBEA/crosslinker, the lower the heat released during the reaction; thus, not enough temperature is achieved for homopolymerization. When DGEBA is too much in excess, the system diffuses the generated heat, and despite still having quite a number of epoxy groups to react, the achieved temperature is not enough. However, D03 formulation seems to exhibit a perfect balance to release enough heat to produce a homopolymerization reaction.

**Figure 4:**
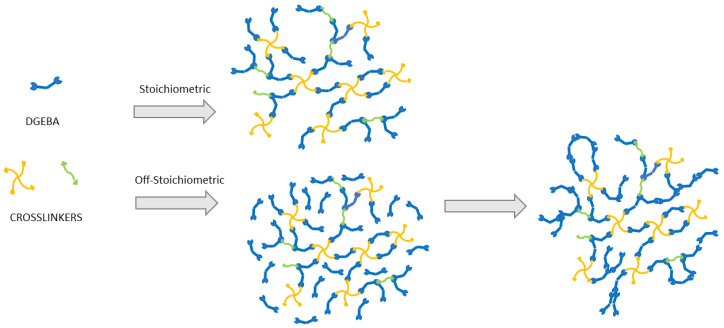
DGEBA epoxy resin, crosslinkers and expected network structures after the stoichiometric and nonstoichiometric curing process.

DGEBA epoxy resin, crosslinkers and expected networks formed after stoichiometric and nonstoichiometric formulations reactions are shown in Figure 4. In case, there are reactive groups left after the room-temperature curing stage; the postcuring of the adhesive would enhance the mechanical properties of the material. Otherwise, the cured formulation would remain unaffected, keeping similar mechanical properties and the same Tg.

### 3.2. Rheological Analysis of Curing Process

The differences detected during the curing process by thermal analysis could imply differences in the rheology of the product, which might affect aspects such as wettability, sagging or working time, among others. Thus, it is necessary to monitor the rheology of the new formulations while curing.

**Figure 5:**
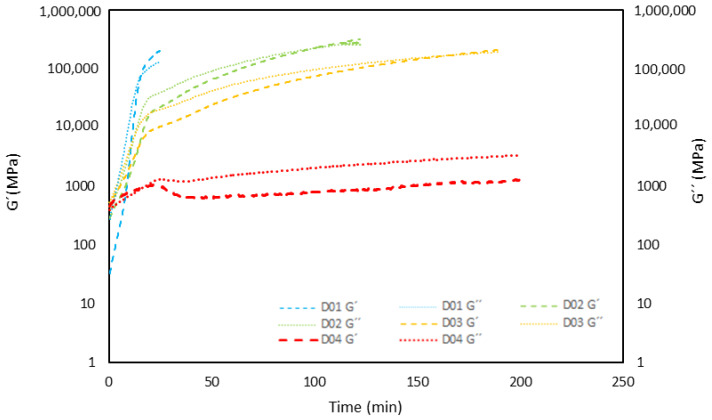
Gel time determination analysis results.

As it can be seen in Figure 5, the higher the DGBEA content in the formulation, the higher the gel time. For the stoichiometric formulation (D01), the gel time is achieved readily, i.e., the reaction occurs very fast, and the material reaches a good consistency (G’ > G”). As the amount of DGBEA increases, a delay in achieving the gel time is detected. The reaction occurs slower; however, it is important to point out that the G’ value achieved is almost identical for the formulations D01, D02 and D03. In these systems, the rheological curves show a two-stage reaction: a first stage with a very fast increase in G’ due to the high reactivity of the components of the curing system; and a second stage, where the increase of G’ becomes slower, that can be attributed to the homopolymerization of the excess of epoxy thanks to the heat released during the first stage. As it can be observed, D04 presented a significant low G’ value, due to the fact that the heat released during the crosslink was not enough to activate the homopolymerization. Since the ratio of the crosslinker is significantly lower, the crosslink density obtained is very low, and thus the G’ value is also minimal.

### 3.3. Thermomechanical Characterization by DMA

DMA analysis was performed for the cure adhesive formulations in order to study the viscoelastic characteristics. If the reaction hypotheses raised up to this point are correct, DMA analysis will show significant changes on Tg.

The structural differences of the studied formulations will have a direct influence on the Tg of the final product. In order to gain insight over the structure built-up for the nonstoichiometric formulations, a DMA characterization was performed on the cured samples after one week kept at room temperature. It should be noted that the D04 mixture did not fully harden at room temperature during the period of one week, and therefore could not be measured.

**Figure 6:**
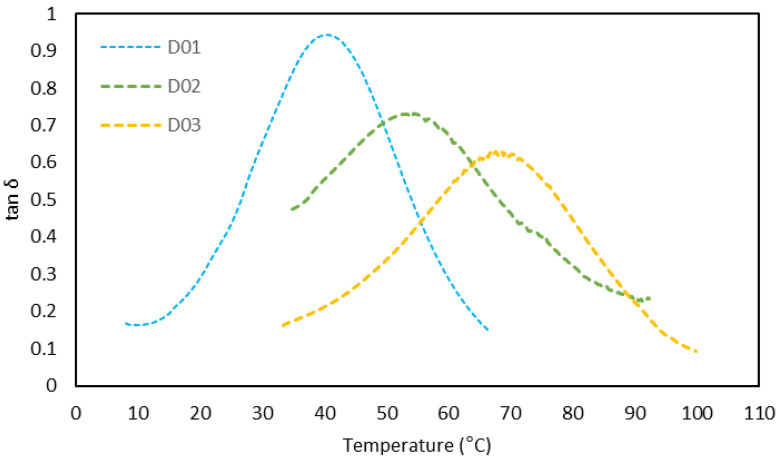
DMA analysis results.

Results obtained showed that as the epoxy resin content is increased in the formulation, the Tg of the final product moves towards higher temperatures without the need to increase the cure temperature (Figure 6). The value obtained for D01 is 40 °C, while for D02 and D03 they are 58 °C and 70 °C, respectively. Thus, an increase of 30 °C in the Tg is achieved by doubling the ratio of epoxy in the formulation. It has to be taken into account that the studied curing reaction takes place at room temperature.

**Figure 7:**
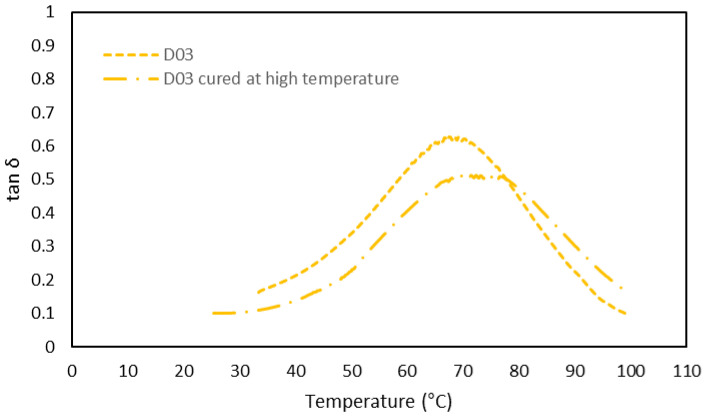
DMA analysis results for room-temperature curing vs. high-temperature curing formulations (80 °C for 2 h and postcuring at 120 °C for 1 h).

As stated before, most of the epoxy formulations developed to obtain high Tg have to go through a high-temperature curing process. In the present case, such a step is not required because further heating does not lead to further reaction. Proof of this is that the sample referenced as D03 was cured at 80 °C for 2 h, followed by a postcuring at 120 °C for 1 h, with almost no change in Tg values obtained (72 °C, Figure 7).

It can be observed by the DMA results obtained (Figure 7) that the variation of Tg is almost insignificant. The peak moves slightly to the right; however, the change observed would not justify the addition of a postcure step for this type of adhesive. The reaction is complete when the adhesive is cured at room temperature, and there is no need to add a post-treatment step, which supposes an added value for this type of product.

### 3.4. Mechanical Characterization

#### 3.4.1. Tensile Strength

**Figure 8:**
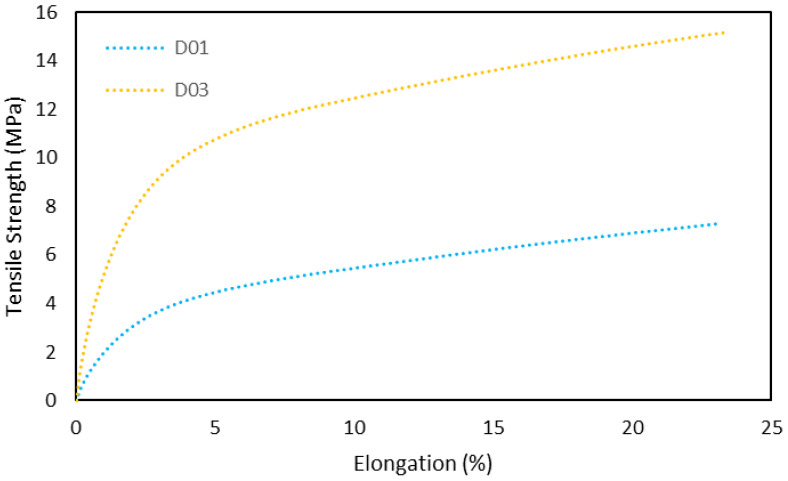
Tensile stress analysis results.

Figure 8 shows representative data from the tensile tests. Apparent differences in stress values can be observed between samples. While D01 adhesive formulation has a tensile strength at break of 5 MPa, D03 adhesive formulation obtains a higher value, reaching 14 MPa. More detailed values can be observed in Table 3. Thus, D03 formulation has a higher ability to resist load under stress or deformation due to the homopolymerization reaction of the excess of the epoxy resin that creates a stiffer polymer network. However, elongation of D01 and D03 formulations remains unchanged. This phenomenon is attributed to the fact that what varies from the stoichiometric to the nonstoichiometric formulation is the epoxy resin quantity, and neither the amount nor the type of crosslinker. The curing system of this adhesive formulation is designed to obtain flexible epoxy adhesives with enhanced toughness. It is noticeable that for the off-stoichiometric formulation, toughness is increased by 56%, thus confirming the structural hypotheses made.

#### 3.4.2. Lap Shear Strength

**Figure 9:**
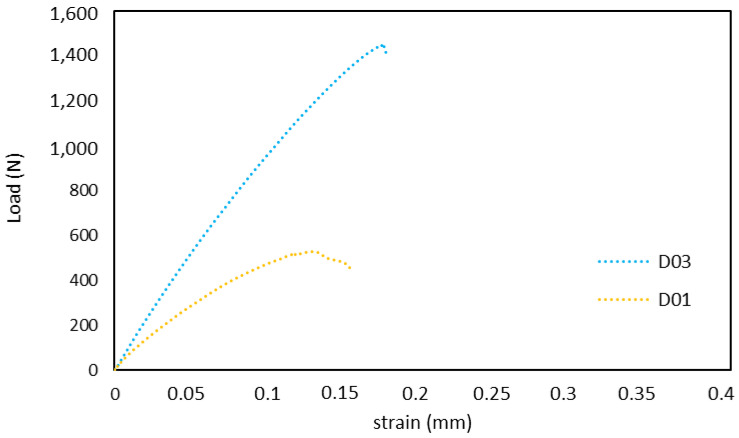
Lap shear bonding test results.

Lap shear adhesion strength of the formulations was also measured in order to evaluate whether the adhesion of the thermoset adhesive varies or not. Both the D01 and D03 adhesive formulations show an adhesive failure. As shown in Figure 9, while the D01 adhesive bond achieves a lap shear strength of 2 MPa, the D03 nonstoichiometric formulation doubles the value. Hydroxyl groups (OH) are responsible for the chemical adhesive property in epoxy groups, and that hydroxyl group concentration influences the durability of the adhesive bond formed between epoxy adhesive and metallic surfaces. Previous literature has also reported that the shear strength depends on the interaction of the functional groups of epoxy with the substrate [34,35]. Thus, more hydroxyl groups indicate a better adhesive property. This is exactly what it is achieved by nonstoichiometric formulation. The higher the epoxy content, the more hydroxyl concentration in the final polymeric structure, as they do not intervene as reagents in the homopolymerization reaction.

## 4. Conclusions

A complex curing system cured at room temperature and based on stoichiometric and nonstoichiometric thiol/amine-epoxy formulations catalyzed by tertiary amines was studied in this work. Firstly, in the presence of an excess of epoxy groups (nonstoichiometric system), thiol-epoxy and amine-epoxy condensation takes place, while the excess of epoxy homopolymerizes in the second stage. The curing rate of the second reaction that takes place at room temperature is much lower than thiol/amine-epoxy condensation reaction curing rate. This difference should be explained by the different reaction mechanism undergone by the two reactions, since on one hand, epoxy-amines and epoxy-thiols suffer a step growth polymerization mechanism that processes rapidly at the start. On the other hand, the excess of epoxy resin undergoes a chain polymerization mechanism catalyzed by the tertiary amine that requires high initiator content in order to be activated rapidly [36,37,38]. The difference in the reaction rate between both processes indicates that it may be possible to separate the two curing stages for custom-tailoring the final material before the homopolymerization reaction finishes.

A ratio between the epoxy resin and crosslinker was optimized to generate enough exotherm to activate the homopolymerization (D03 formulation), which increases the Tg of the product without requiring any post-treatment. This formulation gave rise to an epoxy adhesive formulation with a Tg of 70 °C, maximizing the sustainable operating temperature of the material, and breaking the stablished thumb rule of Tg ≈ Tcure + 25 °C. Regarding mechanical properties, the product developed is tighter and tougher but maintains flexibility of the stoichiometric formulation (D01), which is meant to enhance the resistance to impact-type forces, thermal shock and thermal cycling.

## Figures and Tables

**Table 1:** Formulations and cure schedules.

Sample	Weight Ratio Epoxy/Crosslinker	Cure Time
D01 (Reference)	1:1	r.t. (1 week)
D02	1.5:1	r.t. (1 week)
D03	2:1	r.t. (1 week)
D04	4:1	r.t. (1 week)

**Table 2:** DSC analysis results.

Sample	First Peak		Second Peak	
	ΔHexo (J·g^−1^)	T°max (°C)	ΔHexo (J·g^−1^)	T°max (°C)
D01	127.2	78	74.3	106
D02	99.3	79	79.4	107
D03	76.7	77	10.,7	124
D04	48.8	77	49.5	128

**Table 3:** Results of bulk tensile strength of samples D01 and D03.

Sample	Max. Tensile Strength (MPa)	Elongation	Young’s Modulus (MPa)
D01	4.9 ± 0.63	23.5 ± 1.4	15.8 ± 1.1
D03	14.3 ± 1.5	23.2 ± 2.7	59 ± 2.3

## Data Availability

All relevant data are within the manuscript and available from the corresponding author upon request.

## Supplementary Materials

The following supporting information can be downloaded at https://www.mdpi.com/article/10.3390/polym15020252/s1, Figure S1: Epoxy resin FTIR-ATR spectrum, Figure S2: D001 formulation FTIR-ATR spectrum, Figure S3: D002 formulation FTIR-ATR spectrum, Figure S4, D003 FTIR-ATR spectrum.
