# Supplementary material for: Improving Glass Transition Temperature and Toughness of Epoxy Adhesives by a Complex Room-Temperature Curing System by Changing the Stoichiometry
Source: Polymers (Basel). 2023 Jan 4;15(2):252. doi: 10.3390/polym15020252 (PMC9864282; doi:10.3390/polym15020252)
Supplement: Supplementary file 1 [file polymers-15-00252-s001.zip › polymers-2055047-supplementary.pdf]

Improving Glass Transition Temperature and Toughness of epoxy adhesives by a complex room temperature curing system by changing the stoichiometry

Supplementary Information

Oiane Ruiz de Azua [1], Nuria Agullo [1], Jordi Arbusá [2], and Salvador Borros [1],*

[1] Grup d´Engineyeria de Materials (GEMAT), Institut Quimic de Sarria (IQS), Universitat Ramon Lull, C/Via Augusta 390, 08017 Barcelona, Spain

[2] Sailing Techcnologies, S.L., C/ Calatrava 68, 08017 Barcelona, Spain

** Correspondence: salvador.borros@iqs.url.edu; Tel.: +34932672000*

S1. Epoxy resin FTIR-ATR spectra……………………………………………………………………………………..3

S2. D01 formulation FTIR-ATR spectra ……………………..………………………………………………….4

S3. D02 formulation FTIR-ATR spectra ……………………..……………………..…………………………5

S4. D03 formulation FTIR-ATR spectra ……………………..…………………………………………………6

Figure S1. Epoxy resin FTIR-ATR spectra

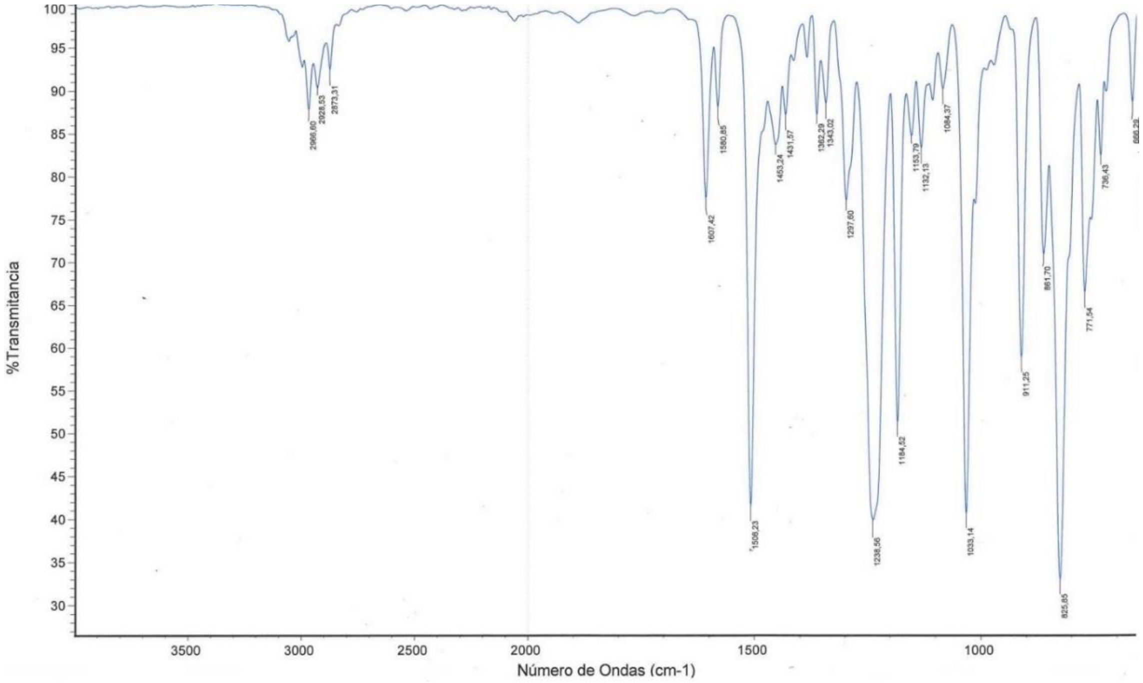

Wave number (cm^{-1})	Assignment / Vibration type
**772, 826**	ArC-H δ
**826, 911**	Epoxy ring δ, Epoxy ring st sim
**1033, 1064**	ArC-O-C aliphatic t sim
**1184**	C-C bond tension between two phenyl groups
**1239**	Aliphatic ArC-O st as, C-O-C as, epoxy ring
**1298**	C-O-C st as
**1362, 1384**	CH_3 sim
**1453**	CH_3 δ as, CH_2 δ
**1508, 1581, 1607**	ArC-C
**2873, 2929, 2967**	C-H bending vibration on methyl groups
3057	C-H in epoxy group, ArC-H st

Two characteristic absorptions of the oxirane ring of epoxy are observed in the range between 3500 cm^{-1} and 700 cm^{-1}. The first one, at 911 cm^{-1}, is attributed to the C-O deformation of the oxirane group. The second band is located at 3057 cm^{-1} approximately and is attributed to the C-H tension of the methylene group in the epoxy ring, although it is also related to asymmetrical and symmetrical C-H stretch of aromatic ring. The presence of these two peaks at the spectre states that epoxy resin has not reacted yet. However, once the epoxy resin starts curing oxirane ring concentration decreases, and this is observed in the spectra as the decrease of the two characteristic absorptions bands, until all oxirane rings are depleted as it can be shown in Figures S2-S4.

Figure S2. D01 formulation FTIR-ATR spectra

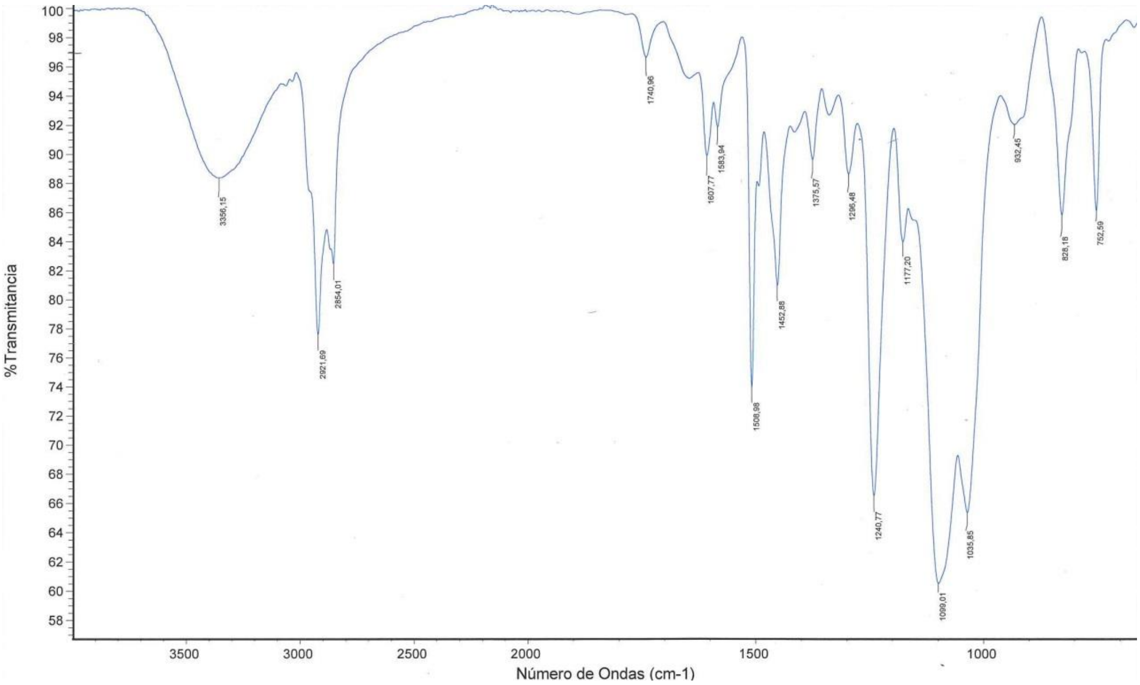

Wave number (cm^{-1})	Assignment / Vibration type
**753,828**	ArC-H δ
**1035**	ArC-O-C alifático t sim
**1099**	C-O-C
**1177**	C-C between two phenyl groups
**1241**	ArC-O-C aliphatic t as
**1296**	C-O-C st as
**1376**	CH_3 δ sim
**1453**	CH_3 δ as, CH_2 δ
**1509, 1584, 1608**	ArC-C
**2854, 2922, 2962**	C-H bending vibration on methyl groups
**3035**	C-H
**3058**	ArC-H st
**3356**	N-H st

Figure S3. D02 formulation FTIR-ATR spectra

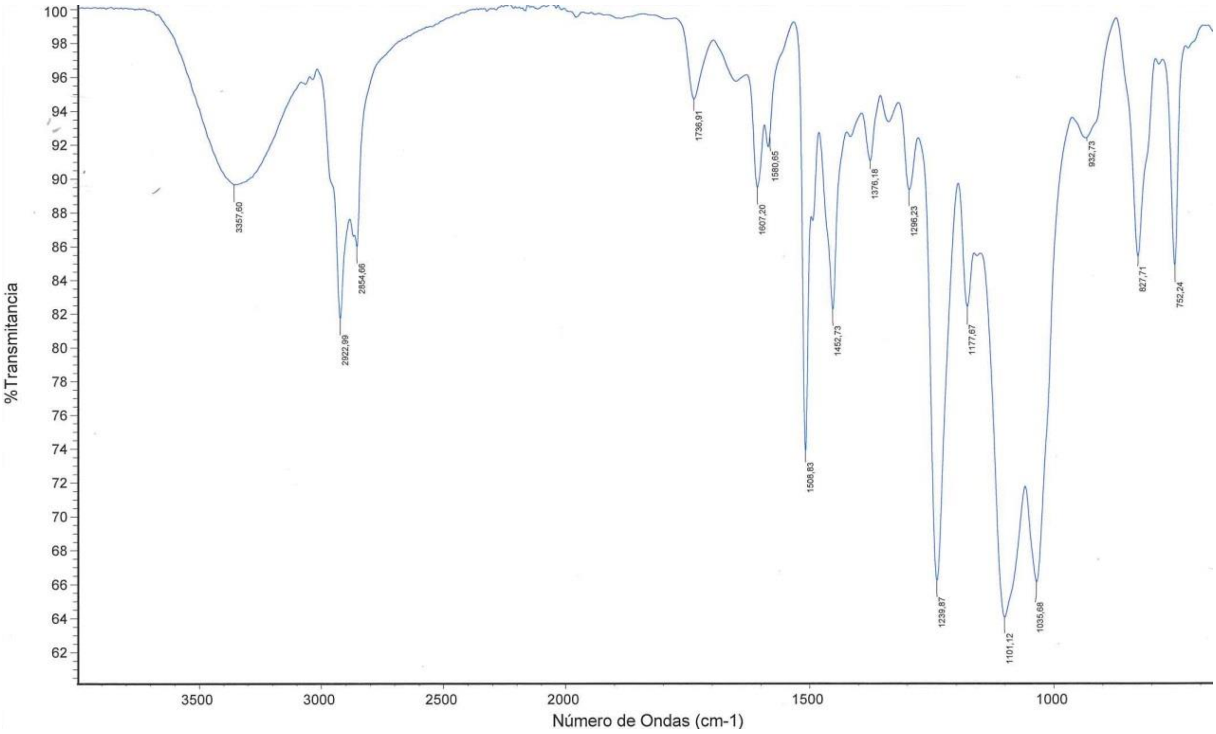

Wave number (cm^{-1})	Assignment / Vibration type
**752,828**	ArC-H δ
**1035**	ArC-O-C alifático t sim
**1101**	C-O-C
**1177**	C-C between two phenyl groups
**1240**	ArC-O-C aliphatic t as
**1296**	C-O-C st as
**1378**	CH_3 δ sim
**1453**	CH_3 δ as, CH_2 δ
**1509, 1581, 1607**	ArC-C
**2854, 2923, 2962**	C-H bending vibration on methyl groups
**3035**	C-H
**3058**	ArC-H st
**3358**	N-H st

Figure S4. D03 formulation FTIR-ATR spectra

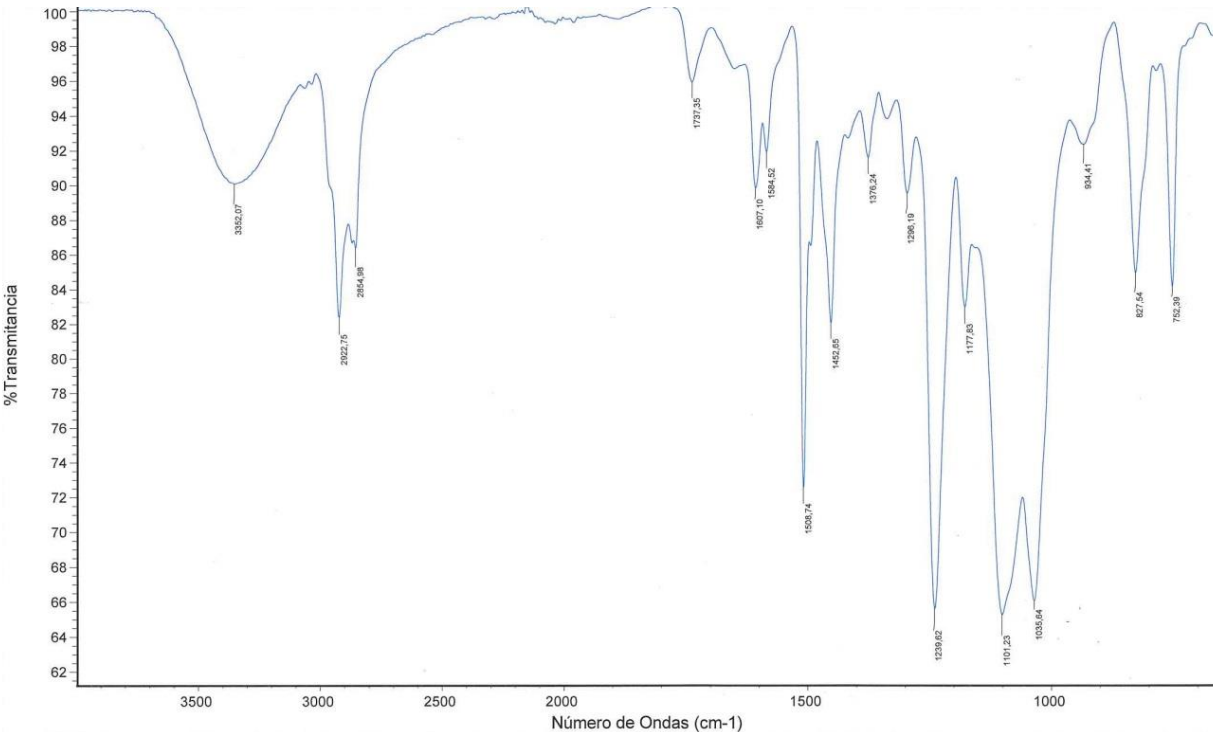

Wave number (cm^{-1})	Assignment / Vibration type
**753,828**	ArC-H δ
**1035**	ArC-O-C alifático t sim
**1101**	C-O-C
**1177**	C-C between two phenyl groups
**1240**	ArC-O-C aliphatic t as
**1296**	C-O-C st as
**1376**	CH_3 δ sim
**1453**	CH_3 δ as, CH_2 δ
**1509, 1585, 1607**	ArC-C
**2854, 2922, 2962**	C-H bending vibration on methyl groups
**3035**	C-H
**3058**	ArC-H st
**3352**	N-H st
